# Experimental immunological demyelination enhances regeneration in autograft-repaired long peripheral nerve gaps

**DOI:** 10.1038/srep39828

**Published:** 2016-12-23

**Authors:** Jun Ge, Shu Zhu, Yafeng Yang, Zhongyang Liu, Xueyu Hu, Liangliang Huang, Xin Quan, Meng Wang, Jinghui Huang, Yunqing Li, Zhuojing Luo

**Affiliations:** 1 Institute of Orthopedics, Xijing Hospital, the Fourth Military Medical University, Xi’an 710032, PR China; 2 The department of anatomy, the Fourth Military Medical University, Xi’an 710032, PR China; 3 General Political Department Hospital of PLA, Beijing 100120, PR China

## Abstract

Peripheral nerve long gap defects are a clinical challenge in the regeneration field. Despite the wide variety of surgical techniques and therapies, autografting is the “gold standard” for peripheral nerve gap reconstruction. The pathological process of Wallerian degeneration from the time of acute injury to efficient regeneration requires several weeks. Regeneration time is critical for nerve reconstruction. Immunological demyelination induced by anti-galactocerebroside antibodies plus guinea pig complement was used to shorten the treatment time. Based on an antigen-antibody complex reaction, the demyelinating agent induced an acute and severe demyelination, leading to the pathological process of Wallerian degeneration during the demyelinating period. This method was used to treat a 12 mm-long sciatic nerve defect in rats. The control groups were injected with one of the demyelinating agent components. The results indicated that anti-galactocerebroside antibodies plus guinea pig complement can significantly shorten treatment time and promote nerve regeneration and functional recovery. In addition, the demyelinating agent can increase the mRNA levels of nerve growth factors and can regulate inflammation. In conclusion, treatment with anti-galactocerebroside antibodies plus guinea pig complement can promote axonal regeneration. This therapy provides a novel method to improve functional recovery in the treatment of long nerve defects.

Peripheral nerve injury (PNI) is an intractable and often enigmatic clinical challenge for surgeons and always leads to functional loss^1^,^2^. Although a variety of surgical techniques and therapies for peripheral nerve regeneration have been developed quickly and comprehensively, the peripheral nerve long gap remains a daunting clinical challenge^3^. The autograft serves as a classic biomaterial and is the ideal treatment for long-distance nerve defects^4^,^5^,^6^,^7^,^8^. Wallerian degeneration is involved in the entire process of nerve repair and creates a microenvironment to support axonal regrowth^9^. Local cell apoptosis and death, axonal necrosis, demyelination, and nerve sheath membrane hyperplasia appear sequentially^10^. However, the pathological processes from the acute injury to an efficient regeneration occur over a relatively long time period. In fact, regeneration time is critical for the patient and determines the quality and outcomes of recovery. Functional recovery during the early phase is beneficial for the recovery of muscle atrophy and sensory malfunction^11^. Consequently, the development of methods to shorten treatment time is a primary concern.

Immunological demyelination is an experimental method used to establish neurologic lesions in animal models, including the multiple sclerosis animal model induced by cuprizone exposure^12^,^13^. We thus proposed that exposure to an appropriate medicament could produce an immunoreaction and thus lead to earlier demyelination in the injured nerve, which would accelerate local cell apoptosis and death. Galactocerebroside (Gal-C), a cerebroside, is abundant in cerebral white matter, the myelin sheath, and kidney. Using Gal-C as an immunoreaction target can induce a local demyelinating response^14^ (Fig. 1s A). Thus, we utilized anti-Gal-C with guinea pig complement as an autoimmune demyelinating agent in our research.

The present study was designed to investigate the effects of immunological demyelination induced by anti-Gal-C antibodies plus guinea pig complement on regeneration in 12 mm autograft-repaired long peripheral nerve gaps in rats. We hypothesized that immunological demyelination (anti-Gal-C antibodies plus guinea pig complement) therapy would shorten the treatment period and increase axonal regeneration. Electron microscopy was used to observe the condition of the axon and the myelin sheath. RT-PCR was conducted to detect markers of the inflammatory reaction and the relationships among inflammatory cytokines, nerve growth factors, and the regeneration process of the target nerve. The fluoro-gold retrograde tracing method was performed to monitor the effects of peripheral nerve regeneration. The sciatic functional index (SFI) was used to evaluate the recovery of motor function. Moreover, histochemical staining of the sciatic nerve and target muscle was used to examine the effects of nerve regeneration. In the current study, we determined that the anti- Gal-C antibody combined with guinea pig complement proteins can shorten the pathological process of Wallerian degeneration in long peripheral nerve gaps and can enhance nerve regeneration.

## Results

### Anti-galactocerebroside Antibodies Plus Guinea Pig Complement Induced Acute Demyelination

**Figure 1:**
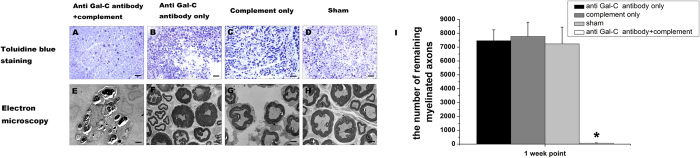
The toluidine blue staining and transmission electron microscope (TEM) images in 1 week end point. In toluidine blue staining, (**A**) the anti-Gal-C antibody and guinea pig complement injection induced a complete demyelination after 1 week. (**B**) The anti-Gal-C antibody injection only group, (**C**) the guinea pig complement injection only group, and (**D**) sham group were not show a complete demyelinating. The same phenomenon was observed under TEM. (**E**) The demyelinating agent made a complete demyelination and the control groups’ agent were not (**F**–**H**). (**I**) The number of remaining myelinated axons were calculated, the result showed that the anti-Gal-C antibody and guinea pig complement injection induced more severe and complete demyelinating than control groups. *P < 0.05 for the anti-Gal-C antibody and guinea pig complement injection group compare with anti-Gal-C antibody injection only group, guinea pig complement injection only group and sham control group. Bar = 50 μm.

Toluidine blue staining and electron microscopy images (Fig. 1) indicated that injections of the anti- Gal-C antibodies and guinea pig complement resulted in widespread and severe edema, inflammation, and Wallerian degeneration in 7 days after nerve injury (Fig. 1A,E). The normal structure of the nerve fibers was nearly undetectable. Light and electron microscopy indicated that the animals in the control injection groups did not exhibit similar demyelination as those in experimental group. The number of remaining myelinated axons also showed that the demyelinating agent induced a severe and complete demyelination in 7 days while the control groups were not (Fig. 1I, P < 0.05). However, signs of Wallerian degeneration such as myelin swelling were found in the lesion regions of the control groups, as revealed by toluidine blue staining and electron microscopy (Fig. 1B–D,F–H). Wallerian degeneration might reflect changes caused by surgery, injection, stitches, and postoperative inflammatory responses.

### Inflammatory Cytokines, Nerve Growth Factors and the Regeneration Process

**Figure 2:**
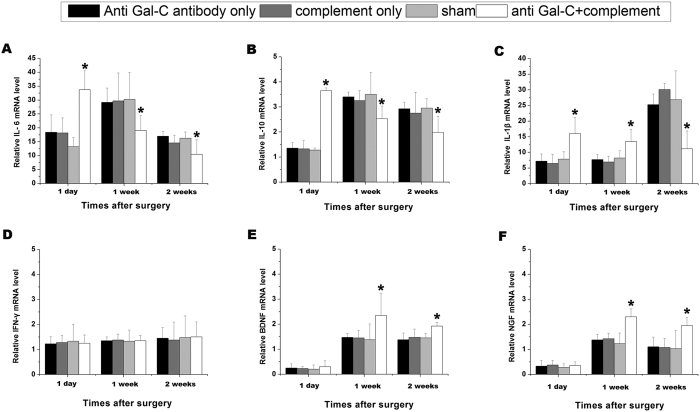
The mRNA levels ofIL-6,IL-10,IL-1β,IFN-γ, BDNF and NGF. (**A**) The relative mRNA level of IL-6 in anti-Gal-C antibody and guinea pig complement injection group was peaked in 1 day and gradually reduced in 1- and 2-weeks. In control groups. The relative mRNA levels of IL-6 were peaked in 1-week point (**B**) The IL-10 mRNA level in anti-Gal-C antibody and guinea pig complement injection group was reach to peak in 1 day while the control groups were peaked in 1-week. (**C**) The relative mRNA level of IL-1β in demyelinating agent injection group was peaked in 1 day. The IL-1β peak appeared in control groups were in 2-weeks. (**D**) The IFN-γ was the only cytokine which is not changed in each time point. (**E**) BDNF and (**F**) NGF were no obvious change in 1 day during all groups. In 1-week and 2-weeks, the relative mRNA levels of BDNF and NGF in anti-Gal-C antibody and guinea pig complement injection group were significantly higher than control groups. all data were expressed as the mean ± standard error of mean (S.E.M). *P < 0.05 for the anti-Gal-C antibody and guinea pig complement injection group compare with anti-Gal-C antibody injection only group, guinea pig complement injection only group and sham control group.

We focused on the mRNA levels of inflammatory cytokines and nerve growth factors at the 1-, 7- and 14-daytime points. In the anti-Gal-C antibody plus complement groups after 1 day, IL-6, IL-10, and IL-1β relative mRNA levels were increased compared to the other three 1-day control groups (Fig. 2A–C, *P < 0.05*). At the 1-week and 2-week time points, IL-6 and IL-10 levels in the experimental group were significantly lower than in the other groups (Fig. 2A and B, *P < 0.05*). The IL-1β level in the experimental group was higher at the 1-week time point and lower at the 2-week time point than in the control groups (Fig. 2C, *P < 0.05*). IFN-γ was the only cytokine with a stable expression pattern among all of the groups at any time point (Fig. 2D, *P* > 0.05). At the 1-day time point, the relative BDNF and NGF mRNA levels were low. At the 1-week and 2-week time points, the NGF and BDNF mRNA levels in all groups were significantly increased (Fig. 2E and F, 1-day point, *P* > 0.05). In addition, the levels of the two nerve growth factors in the experimental group were higher than in the control groups (Fig. 2E and F, 1week and 2weeks, *P < *0.05). To summarize, IL-6, IL-10, and IL-1β relative mRNA levels in the anti-Gal-C antibody plus complement group peaked at an earlier time after surgery and expression gradually declined over several weeks. Compared with the experimental group, the anti-Gal-C antibody injection-only group, the complement injection-only group and the sham group exhibited a different expression pattern of IL-6, IL-10, and IL-1β mRNA. IL-6 and IL-10 mRNA levels in control groups remained at a relatively low level at day 1 and increased at the 1-week time point. mRNA levels were then lower at the 2-week time point. The relative mRNA level of IL-1β differed from the levels of IL-6 and IL-10 in the control group. At the 1-day and 1-week time points, IL-1β levels remained low. At the 2-week time point, the level of mRNA was significantly increased. The BDNF and NGF levels in all groups peaked at approximately the 1-week time point and then declined at the 2-week time point. The above expression pattern may be due to the effects of various inflammatory cytokines induced by the immunoreaction after the injection of the demyelinating agent.

### Immunological Demyelination Enhances Axon and Myelin Sheath Regeneration

**Figure 3:**
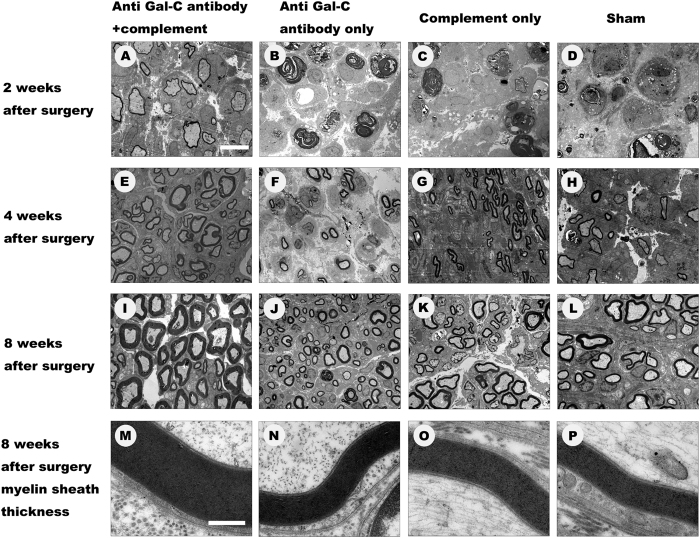
The representative TEM images of regenerated axons and myelin sheath. The regeneration effects of anti-Gal-C antibody and guinea pig complement injection in (A)2-(E)4- and (I)8-weeks after surgery significant better than anti-Gal-C antibody injection only group (**B**,**F**,**J**,**N**), guinea pig complement injection only group (**C**,**G**,**K**,**O**) and sham control group (**D**,**H**,**L**,**P**). Bar for **A** to **L** = 10 μm, bar for **M** to **P** = 500 nm.

To analyze the effect of the demyelinating agent on axonal regeneration, we used an electron microscope to determine the differences among the groups. A 1 mm section of the sciatic nerve distal to the incision was chosen and evaluated at the 2-, 4-, and 8-week time points (Fig. 3). However, we found that some newly formed axons appeared only in the anti- Gal-C antibody and guinea pig complement injection group (Fig. 3A). Samples from animals in the anti- Gal-C antibody injection group, the guinea pig complement injection group, and the transplant surgery group exhibited apoptosis and complete disintegration of axonal structures as opposed to regenerated axons (Fig. 3B–D). These results were similar in the anti-Gal-C antibody and guinea pig complement injection group at the 1-week time point (Fig. 1E). The results demonstrate that the demyelinating agent initiated the axon and myelin disintegrating process at least 7 days earlier. At the end of the 4-week time point, more regenerated axons appeared in the experimental group compared to the other three control groups (Fig. 3F–H). These regenerated axons appeared to be more complete and mature (Fig. 3E). At the end of the 8-week time point, massive axons with Schwann cells were observed in samples from the anti-Gal-C antibody and complement protein injection group (Fig. 3I). The quality and quantity of axons in the control groups were much lower than in the experimental group (Fig. 3J–L). We also focused on the myelin sheath thickness in all groups at the 8-week time point (Fig. 3M–P). As observed in the ultra-thin tissue sections, the experimental group myelin sheath thickness was higher in the experimental group than in the control group.

**Figure 4:**
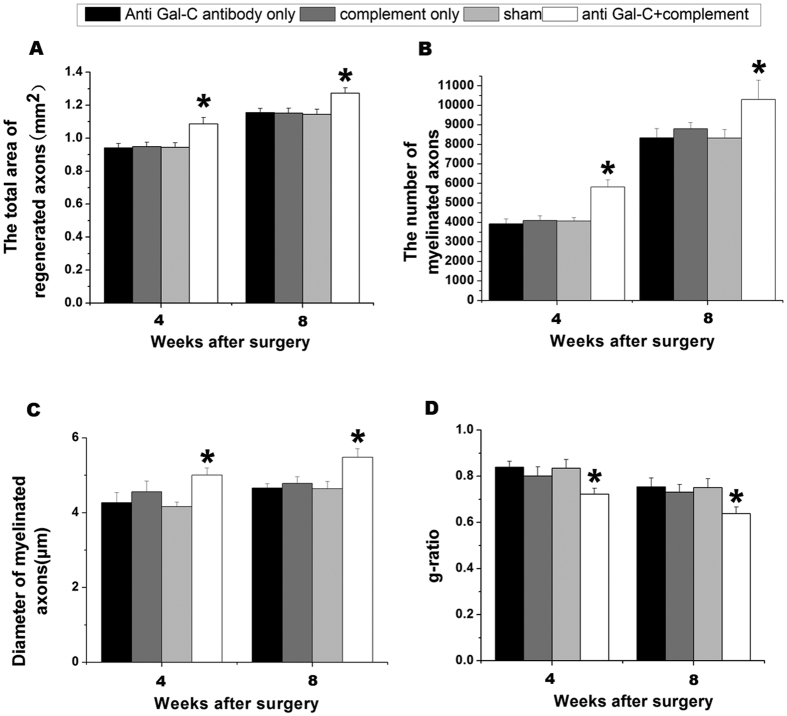
Morphometric evaluations of regenerated nerves. (**A**) The cross-sectional area of regenerated nerve, (**B**) quantification of the myelinated axons, (**C**) the diameter of myelinated axons, and (**D**) G-ratios in the distal end from the distal incision 1 mm of operated sciatic nerves. The anti-Gal-C antibody + complement injection was obviously better than control groups in nerve regeneration, from quantity to quality. All data were expressed as the mean ± standard error of mean. *P < 0.05 for the anti-Gal-C antibody and guinea pig complement injection group compare with anti-Gal-C antibody injection only group, guinea pig complement injection only group and sham control group.

We calculated the total area of regenerated axons, the number of myelinated axons, the diameter of myelinated axons, and the G-ratio at the end of the 4-and 8-week time points in all groups (Fig. 4). The total area of regenerated axons in the anti-Gal-C antibody and complement protein injection group was 1.08 ± 0.03 mm^2^ at 4 weeks and 1.27 ± 0.03 mm^2^ at 8 weeks, which was significantly higher than in the anti-Gal-C antibody injection-only group (0.94 ± 0.02 mm^2^ at 4 weeks and 1.15 ± 0.02 mm^2^ at 8 weeks), the guinea pig complement injection-only group (0.95 ± 0.02 mm^2^ at 4 weeks and 1.15 ± 0.02 mm^2^ at 8 weeks) and the group that only received the transplant surgery (0.94 ± 0.02 mm^2^ at 4 weeks and 1.14 ± 0.03 mm^2^ at 8 weeks)(Fig. 4 above left, *P < 0.05*). The number of myelinated axons exhibited the same trend. The values in the experimental group ({5.81 ± 0.38} × 10^3^ at 4 weeks and {10.30 ± 0.99} × 10^3^ at 8 weeks) were generally higher than in the control groups (the anti-Gal-C antibody injection-only group: {3.93 ± 0.25} × 10^3^ at 4 weeks and {8.33 ± 0.48} × 10^3^ at 8weeks; the guinea pig complement injection-only group: {4.09 ± 0.24} × 10^3^ at 4 weeks and {8.79 ± 0.33} × 10^3^ at 8weeks;and the sham: {4.06 ± 0.19} × 10^3^ at 4 weeks and {8.32 ± 0.44} × 10^3^ at 8 weeks) (Fig. 4 above right, *P < *0.05). The diameter of myelinated axons was also measured. The experimental group exhibited diameters of 5.00 ± 0.20 μm at 4 weeks and 5.48 ± 0.23 μm at 8 weeks (Fig. 4 below left, *P < *0.05). Additional analyses showed that the degree of myelination (G-ratio) in the experimental group was better than which in the three control groups; Compared with each control group, The anti-Gal-C antibody and complement injection group G-ratios were more approximate to 0.6 (a normal nerve G-ratio) at 4 weeks (0.72 ± 0.02) and 8 weeks (0.63 ± 0.03) (Fig. 4 below right, *P < 0.05*).

We also observed electron microscopy photo of naïve rats’ sciatic nerve to compare with the operation side nerve electron microscopy photo of anti-Gal-C antibody with complement injection group in 8 weeks (Fig. 3s A and B). The anti-Gal-C antibody and complement injection group G-ratio was approximate to naïve rats (Fig. 3s C). However, the quantity of axons in anti-Gal-C antibody + complement injection group was satisfactory but it does not reach to normal ones (Fig. 3s D).

### Immunological Demyelination Accelerates Motor and Nerve Function Recovery

**Figure 5:**
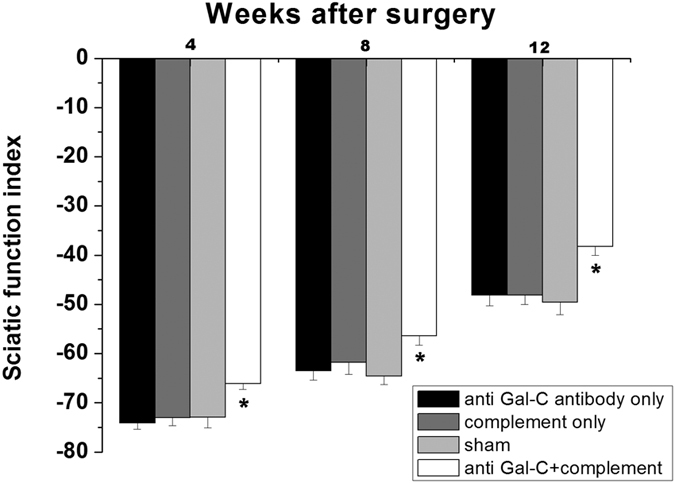
The sciatic function index (SFI) in each group. The results showed that the demyelinating agent improved the SFI obviously in 4-, 8- and 12-weeks. All data were expressed as the mean ± standard error of mean. * P < 0.05 for the anti-Gal-C antibody and guinea pig complement injection group compare with anti-Gal-C antibody injection only group, guinea pig complement injection only group and sham control group.

The sciatic functional index (SFI) measurements were performed at the 4-, 8- and 12-week time points. Compared to the three control groups, the anti-Gal-C antibody with complement injection group exhibited a stronger recovery in sciatic nerve function. At the 4-, 8- and 12-week time points, the SFI values of the experimental group (−66.12 ± 3.38 at 4 weeks,−56.39 ± 5.63 at 8 weeks and −38.23 ± 5.35 at 12 weeks) were significantly better than in the control groups (Fig. 5, *P < *0.05). These results indicated that injection of the demyelinating agent enhanced motor function recovery.

**Figure 6:**
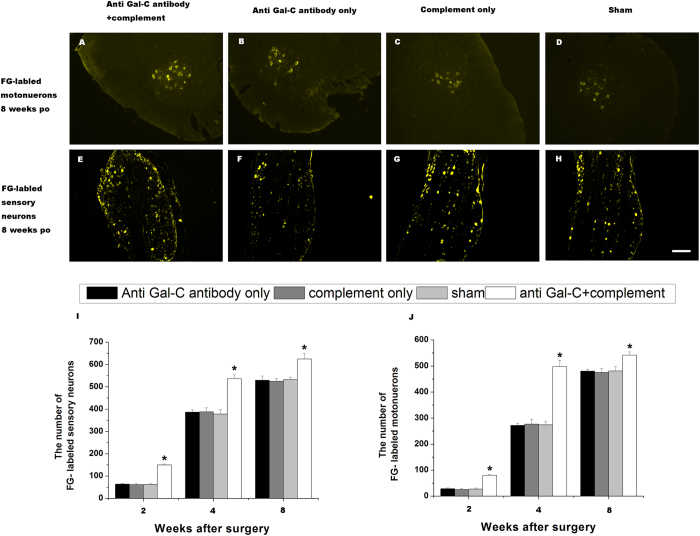
Fluoro-Gold (FG) retrograde tracing in each group. The representative images of FG-positive motoneurons in spinal cord (**A**,**B**,**C**,**D**) and sensory neurons in DRG (**E**,**F**,**G**,**H**) in the anti-Gal-C antibody and guinea pig complement injection group (**A**,**E**), anti-Gal-C antibody injection only group (**B**,**F**), guinea pig complement injection only group (**C**,**G**) and sham control group (**D**,**H**) were shown in 8 weeks after surgery. The average number of FG-positive sensory neurons and motoneurons in each group were shown in (**I**) and (**J**) respectively. In anti-Gal-C antibody and guinea pig complement injection group, the FG-positive motoneurons in spinal cord and sensory neurons in DRG were significantly higher than control groups. All data were expressed as the mean ± standard error of mean. *P < 0.05 for the anti-Gal-C antibody and guinea pig complement injection group compare with anti-Gal-C antibody injection only group, guinea pig complement injection only group and sham control group. Bar = 250 μm.

**Figure 7:**
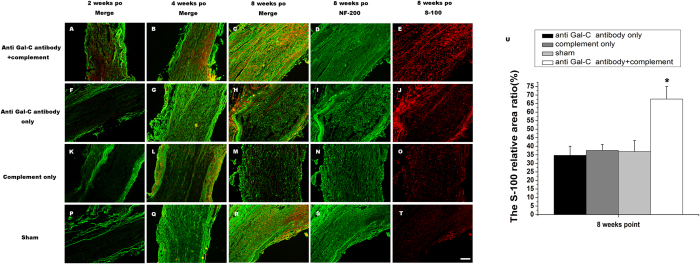
Double-immunohistochemical staining for NF-200 and S-100. The representative merged images of regenerated nerves in the midst of left sciatic nerve in the anti-Gal-C antibody and guinea pig complement injection (**A**,**B**,**C**), anti-Gal-C antibody injection only group (**F**,**G**,**H**), guinea pig complement injection only group (**K**,**L**,**M**) and sham control group (**P**,**Q**,**R**,**S**,**T**) at 2 -(**A**,**F**,**K**,**P**) 4-(**B**,**G**,**L**,**Q**) and 8 weeks (**C**,**H**,**M**,**R**) after surgery. The NF-200 (**D**,**I**,**N**,**S**) and S-100(**E**,**J**,**O**,**T**) staining images in 8 weeks end point were present respectively.(U) The S-100 relative area ratio in graft nerve at 8 weeks point. In 2-, 4- and 8-weeks, the nerve fibers (red fluorescent) and Schwann cells (green fluorescent) regeneration in demyelinating agent injection group were obviously better than those in control groups. The S-100 relative area ratio at 8 weeks in anti-Gal-C antibody and guinea pig complement injection group showed a higher rate than control groups. All data were expressed as the mean ± standard error of mean. *P < 0.05 for the anti-Gal-C antibody and guinea pig complement injection group compare with anti-Gal-C antibody injection only group, guinea pig complement injection only group and sham control group. Bar = 250 μm.

Due to the low number of fluoro-gold (FG)-labeled cells in all groups at the 1-week time point, the FG retrograde-labeling analysis was performed in samples at 2-, 4-, and 8-week time points. FG-positive cells were quantified in the ventral horn of the spinal cord (motor neurons) and in the dorsal root ganglion (DRG, sensory neurons) in all groups (Fig. 6). The number of FG-labeled sensory neurons and motor neurons in the anti-Gal-C antibody with complement protein group was much higher than in the control groups (Fig. 6I and J, *P < *0.05). As shown in Fig. 7A–H, treatment with the demyelinating agent was associated with an increased number of sensory neurons and motor neurons that survived and regenerated into the distal stumps.

### Morphology

NF-200/S-100 double-labeled immunofluorescence analysis was performed to assess the effects of the demyelinating agent on neural morphology. The NF-200-positive cells were reflected the distribution of the neurofilament protein, and S-100-labeled cells represented the Schwann cells. We focused on the middle portions of the nerve samples. At the 2-week time point, NF-200/S-100 double-labeled cells in the anti-Gal-C antibody with complement group exhibited healthier morphology than cells in the control groups (Fig. 7A,F,K,P). The same phenomenon was observed in samples at the 4-week time point, from nerve regeneration to Schwann cell migration (Fig. 7B,G,L,Q). In samples from the last observation point (8 weeks), cells in the experimental group exhibited a healthier morphology than cells in the control groups (Fig. 7C,H,M,R). NF-200 staining showed that the neurofilament proteins in the 8-week experimental group exhibited a healthier morphology and directionality (Fig. 7D,I,N,S). S-100 staining also indicated enhanced Schwann cell regeneration and migration in the anti-Gal-C antibody with complement injection group (Fig. 7E,J,O,T). We analyzed the S-100 relative area ratio in graft nerve at 8 weeks point, the experimental group nerve present a higher rate in Schwann cells compared with control groups (Fig. 7U, P < 0.05).

**Figure 8:**
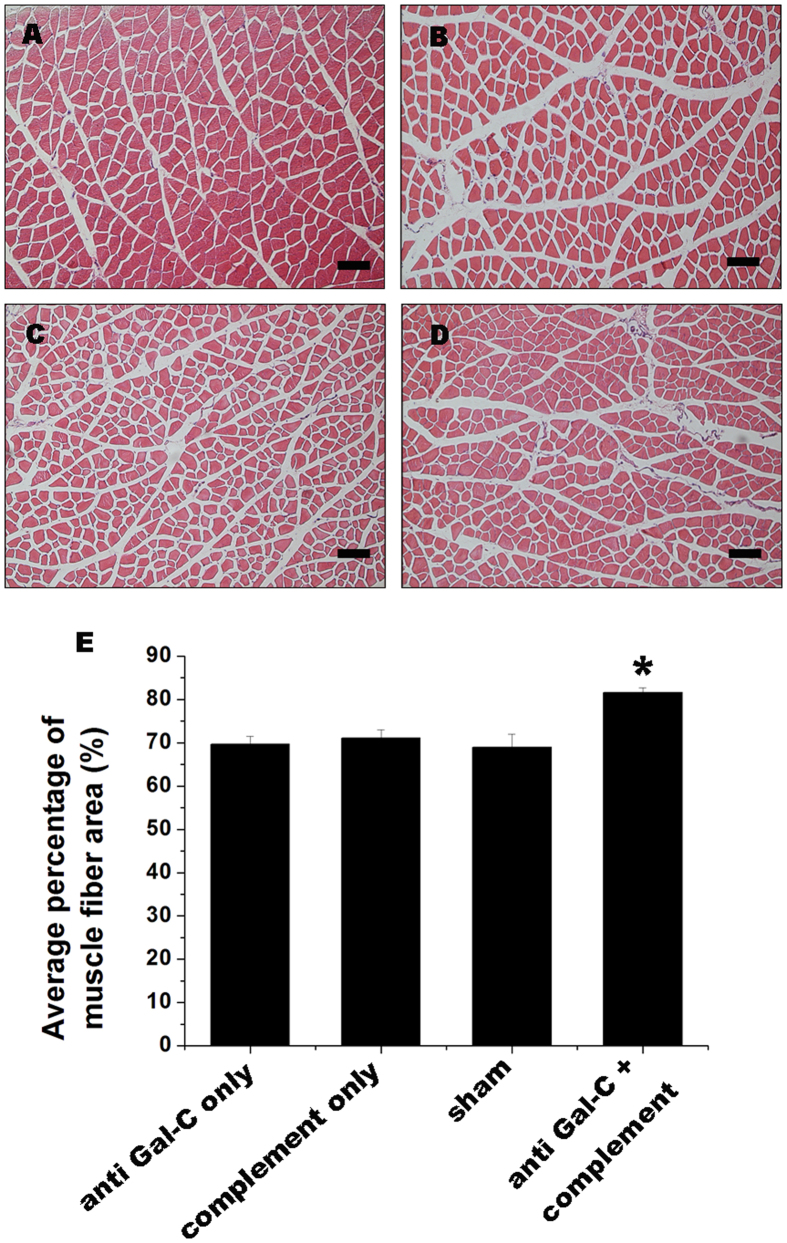
Histological analysis of target muscle in HE staining. Representative light micrographs of the transverse-sectioned gastrocnemius muscle following HE staining for the operated limb in the (**A**) anti-Gal-C antibody and guinea pig complement injection group, (**B**) anti-Gal-C antibody injection only group, (**C**) guinea pig complement injection only group and (**D**) sham control group in the end of 8 weeks after surgery.(**E**) the average percentage of muscle fiber area. The eosin positive area (red) in anti-Gal-C antibody and guinea pig complement injection group was significantly higher than control groups. All data were expressed as the mean ± standard error of mean. *P < 0.05 for the anti-Gal-C antibody and guinea pig complement injection group compare with anti-Gal-C antibody injection only group, guinea pig complement injection only group and sham control group. Bar = 50 μm.

The morphology of gastrocnemius muscles was examined to evaluate the effects of treatment with the demyelinating agent on functional motor recovery. Animals in the demyelinating agent injection group (Fig. 8A–D) exhibited better functional muscle recovery than other groups. In addition, the average percentage of target muscle fiber area of the anti-Gal-C antibody plus complement group (81.59 ± 1.15%) was significantly higher than in the anti-Gal-C injection-only group (69.66 ± 1.81%), the complement injection-only group (71.12 ± 1.88%), and the sham group (69.01 ± 3.01) (Fig. 8E, *P < *0.05). These results suggested that the demyelinating agent could promote motor function recovery of the injured limb.

## Discussion

Despite the development of several surgical techniques^15^, new bridging materials^16^,^17^,^18^,^19^, and therapies^20^,^21^,^22^,^23^,^24^, the autologous nerve graft is still the “gold standard” in long peripheral nerve gap reconstruction. Although there are some disadvantages to the procedure, such as a lack of autologous nerve sources or feeling obstacles for providing area during surgery, the autologous nerve graft is closest to the normal physiological structure and function when the above-noted difficulties can be overcome. After a peripheral nerve defect injury, regeneration time and quality are crucial for all patients. Thus, approaches to shorten the treatment time and improve the quality of regeneration are of vital importance.

Classical pathological changes after nerve injury include axon necrosis, myelin disintegration, and nerve sheath membrane hyperplasia at the distal end of the nerve. The fragments of degenerated myelin and axonal debris are phagocytosed by macrophages. Schwann cells then promote reconstruction and migration to rebuild the nerve regeneration microenvironment with basement membranes. This process was described and defined by Waller in 1850. However, Wallerian degeneration proceeds over 7–9 days to complete the axon necrosis procedure and 12–15 days to complete myelin disintegration, after which the repair period can begin. In other words, despite the effects of inflammation and edema, nerve regeneration is ongoing at 2-3 weeks after injury. Thus, a relatively long time is required for neural restoration and functional target organ recovery. And it is very adverse for a long peripheral nerve gap patient.

An immunoreaction was performed through a complex and synergistic process caused by the effects of antigen, antibody, immune molecules, immune cells and immune tissues. Nerve demyelination, however, is a controversial choice for clinicians. Indeed, widely demyelinating reactions can result in neuropathological changes in peripheral nerve repair and can block nerve regeneration. However, a local demyelination induced by anti-Cal-C antibody with complement does not cause a sizable reaction; this technique was safe for nerve regeneration. The acute and damaging immunoreaction was always considered an adverse factor. Ydens claimed that acute peripheral nerve injury triggers an anti-inflammatory and immunosuppressive response, and the authors presumed that neurodegeneration-induced immune responses may lead to nerve regeneration therapy, what is inspire us^25^.

Previous studies demonstrated that the injection of a demyelinating agent in the epineurium improved peripheral nerve regeneration following transection injury or crush injury^26^,^27^. In contrast to agents used in previous studies, a 1:1 ratio of anti-Gal-C antibody and guinea pig complement without PBS was used in our experiment. The reason for using this formulation was to avoid complement protein degradation and destruction in the process of preparing the solution. Furthermore, the use of a relatively pure reagent mixture can rule out interference from other factors. However, according to the results of toluidine blue staining and electron microscopy, our demyelinating agent played the same role in treatment. An acute and thorough immune demyelinating reaction had occurred by 1week after agent injection, and nerve regeneration was observed within 2 weeks. The anti-Gal-C antibody with complement injection can shorten the pathological process of Wallerian degeneration in long peripheral nerve gaps. The demyelinating agents enhanced axon disintegration and demyelinating reactions at early time points after surgery. We compared transmission electron microscopy images in the 1-week experimental group with the 2-week control groups and found that the pathological reactions of the experimental group such as inflammation and edema were attenuated compared to the control groups. This finding may be due to the protective pathologic changes induced by the acute immune response. More immune cells cleared more cellular debris. The processes of axon necrosis and demyelination were faster than in control groups. Interestingly, the complement, which can induce an immune response due to host antigen-antibody complexes, did not cause an acute immune response in the complement injection control group. It may due to the relative lack of sufficient antigen-antibody complexes to combine with the complement.

Inflammatory cytokines and two nerve growth factors were evaluated at the 1-day, 1-week and 2-week time points to characterize the degree of local inflammationat early regeneration times. At the 1-day time point, the anti-Gal-C antibody plus complement agent increased levels of IL-6, IL-10, and IL-1β in the injured nerve. This result demonstrated that the demyelinating agent induced an acute and severe immuno-inflammatory response promptly after injection. The other groups, however, exhibited inflammatory cytokines that peaked at the 1-week time point with the exception of IL-1β. This finding indicated that severe and extensive immunoreactions were delayed in the control groups. IL-1β mRNA peaked at the 2-week time point, which may be due to the distinct function of IL-1β during inflammation. IFN-γ was the only cytokine that was relatively unchanged in all of the groups and at all of the time points. This finding may be due to the fact that IFN-γ is associated with an M1 phenotype of macrophages and is not activated in acute nerve injury. In addition, differences in the expression of two nerve growth factors in the experimental group and in the three control groups indicated that the anti-Gal-C antibody plus complement injection promoted nerve regeneration in long gap injury. It is may be due to the anti-Gal-C antibody and guinea pig complement injection induced a severe inflammatory reaction, pushed on process in Wallerian degeneration and made the best regeneration internal environment appeared in advance.

From the nerve to the target muscles, significant differences were found in the anti-Gal-C antibody plus complement injection group compared to the three control groups. The number of regenerated axons, the mean diameter of regenerated axons, the total area of regenerated axons and the G-ratio of experimental animals were all significantly different from values in the control groups at all-time points. This result is in striking contrast to the axonal regeneration at the 8-week time point under transmission electron microscopy. Regardless of the axon number and the thickness of the myelin sheath, electron microscopy photos of the experimental group were visually compelling. These findings suggest that anti-Gal-C plus complement therapy can enhance nerve regeneration via the modulation of a demyelinating reaction. Compared with naïve rats, the anti-Gal-C antibody and complement injection presented a satisfactory regeneration effect in nerve quality. However, the demyelinating agent injection cannot reach to normal level in axon quantity and SFI in 8-weeks. Nerve regeneration after long gap injury is an intricate process. Even after several months of autografting reconstruction, the function of injured limb cannot reach to normal. In this study, we mainly focused on whether the demyelinating agent can optimize the autografting surgery, and then accelerate nerve defect recovery in early stage. Obviously, the results showed that the injection of anti-Gal-C antibody + complement is helpful for nerve gap injury regeneration within 12 weeks. Our data indicate that 2 months after receiving demyelinating agent therapy, regeneration of the injured sciatic nerve increased by 20–33%. FG-labeled cells in samples from the 2-week time point were very rare in all control groups. This finding was determined to be due to poor recovery without therapy. The numbers of FG-labeled motor neurons and sensory neurons in the anti-Gal-C antibody plus complement group were significantly higher than in the anti-Gal-C antibody injection-only group, the complement injection-only group and the sham group. These data indicated that more axons were successfully regenerated into the distal stumps and the spinal cord anterior horn motor nerve. The NF-200/S-100 double-labeled immunohistochemical staining images showed that anti-Gal-C antibody plus complement injection can promote the regeneration and orientation of neurofilament protein and Schwann cells. SFI and HE staining of muscles in the experimental group demonstrated that functional outcomes were improved at every test point. In brief, our demyelinating agent injection promoted nerve regrowth and enhanced target muscle functional recovery.

Whether the anti-Gal-C antibody or complement can promote other processes such as the activation of cellular pathways, however, it is still unclear. We hypothesize that the effects of the anti-Gal-C antibody plus complement agent were mainly due to acute and complete immunoreaction.

In conclusion, the anti-Gal-C antibody plus complement agent can promote axonal regeneration and functional recovery after long peripheral nerve defect. In addition, the acute immunoreaction induced by the demyelinating agent can reduce the inflammation response. This effect provides a favorable microenvironment for axonal regeneration, which is beneficial for nerve regeneration and functional recovery. Thus, demyelination therapy can be used in improving nerve regeneration after long peripheral nerve gap injury. This therapy may solve the problem of the recovery time associated with autologous nerve transplantation and provides a new method for the treatment of clinical long nerve segmental defects.

## Methods

### Animals

Adult female Sprague-Dawley rats weighing 230 to 250 g were used in the experiments. All of the animals were provided by the laboratory animal center of the Fourth Military Medical University. Our research was conducted in accordance with the Guide for Care and Use of Laboratory Animals published by the US National Institutes of Health (NIH Publication No. 85-23, revised 1996) as adopted. All experimental protocols were approved by the Review Committee for the Use of Human or Animal Subjects of Xijing Hospital, The Fourth Military Medical University. Animals were anesthetized with 1% pentobarbital solution during surgery (0.4 ml/100 g body weight). The surgical method and the demyelinating agent injection were standardized. All of the injection solution was prepared at a clean bench. In total, 256 rats were randomized into 4 groups of 64 animals for each (Fig. 2s). All of the animals in each of the groups were treated with the surgical procedures and the agent injection as described below.

### Surgical Procedures

After disinfection with an iodophor solution and a solution of 75% alcohol, the left sciatic nerve was exposed by a skin incision and a cut in the underlying muscles in the left lateral thigh. A 12 mm sciatic nerve segment was excised and removed. The removed nerve segment was rotated by 180° and transplanted in the injured area with a 10-0 epineural suture of monofilament polyamide under 40 × magnifications^28^ (Fig. 1s B). The skin was closed with 6-0 stiches.

### Agent Injection

A demyelinating agent was injected into the distal area of the sciatic nerve graft with a silicon-coated glass micropipette attached to a Hamilton syringe in anti-Gal-C antibody with guinea pig complement group (n = 64). The demyelinating agent consisted of an anti-Gal-C antibody (Millipore, AB142) and guinea pig complement (SIGMA) at a ratio of 1:1. All of the animals received a total volume of 5 μl of drugs with same doses as in all groups (physiological saline was used to maintain a constant volume). Control injection groups consisted of anti-Gal-C antibody only (n = 64), guinea pig complement only (n = 64), and a sham control group that only received the transplant surgery (n = 64).

### Toluidine Blue Staining and Electron Microscopy

Samples from each group of rats were subjected to toluidine blue staining and electron microscopy at 1-, 2-, 4-, and 8-week time points. Animals were killed via pentobarbital injection (200 mg/kg). The left sciatic nerves were fixed in 4% glutaraldehyde in 0.1 M phosphate buffer (pH 7.4). Sections of the sciatic nerves containing autografts were cut into 1 mm transverse blocks. The tissues were postfixed in 1% OSO_4_ in 0.1 M sodium cacodylate buffer (pH 7.3) for 1 hour at room temperature, dehydrated in ethanol, and embedded in resin. One micrometer-thick sections were cut and stained with alkaline toluidine blue. All slices were examined by light microscopy (AH3; Olympus). For electron microscopic analysis, tissue sections were cut at a thickness of100 nm and were mounted on copper grids, stained with uranyl acetate and lead citrate, and observed under a transmission electron microscope (H-600; HITACHI).

### Axon and Myelin Sheath Thickness Quantification

Axon regeneration was estimated by the total number of myelinated axons per section at 2000 × magnification, the total area of regenerated nervesper semi-thin section, and the mean diameter of nerve fibers and was analyzed at the end of the 4-and 8-week time periods. Remyelinated axons were identified by the characteristically thick myelin sheaths relative to the diameter of the axons. The number of demyelinated axons was also recorded, especially at the1-and 2-week time points. G-ratios (myelin sheath thickness/axon diameterper ultra-thin section) reflect the degree of myelination. Random sampling was performed, and G-ratios were calculated at 20,000 × magnification. Measurements of myelin sheath thickness were taken using a transmission electron microscope (H-600; HITACHI).

### RT-PCR

In brief, RT-PCR was performed according to the manufacturer’s instructions. Total RNA was isolated from distal regenerated nerve segments with the RNeasy^TM^ kit (Qiagen, Valencia, CA) at 1-day and 1- and 2-week time points. Then the reverse transcription step using 1 ng of RNA was performed with the PrimeScript RT Master Mix kit (Takara, Japan), β-actin as an internal control. The PCR reaction was then executed using a SYBR Premix Ex Taq II (Takara, Japan). All RT reactions were run in triplicate in a GeneAmp PCR 9700 Thermocycler (ABI); the reaction mixture was heated to 95 °C for 2.5 min and then amplified for 40 cycles as follows: 95 °C for 35 s (denaturation), 54 °C for 30 s (annealing), and 65 °C for 5 s (extension)^29^,^30^. Forward and reverse primer pairs with annealing temperatures are described in Table 1s.

### Fluoro-gold Retrograde Tracing

Retrograde labeling was performed immediately following agent injection (in Groups A, B, and C) or surgical procedure (Group D), and the tissue was harvested at the 1-week time point. The same operation was performed at 2, 4, and 8 weeks after surgery. One week before the tissue was harvested, the injured sciatic nerves were injected with 2 μl of 4% FG (Biotium Inc., CO) solution at a location 2 mm distal to the grafts, and then the incision was sutured. After 7 days, the animals were euthanized. The rats were perfused with 4% paraformaldehyde in 0.1 M PBS. The L4, L5, and L6 segments of the lumbar spinal cord and the same segments of the left-side dorsal root ganglion (DRG) were harvested. All tissue samples were postfixed in 4% paraformaldehyde for 24 h andcryoprotected in 30% sucrose overnight at 4 °C, after which frozen sections were obtained. The spinal cords were sectioned at a thickness 25 μm, and the DRG was sectioned at a thickness of 15 μm. All of the sections were attached to poly-lysine-precoated glass slides and were viewed and photographed under a fluorescent microscope (BX-60; Olympus). According to the method of Abercrombie, the split cells were counted twice. The number of FG-labeled spinal cord motor neurons was counted by 2 independent investigators who were blinded to the experimental groups.

### Motor Function Evaluation

The walking track analysis was performed at 4, 8, and 12 weeks after surgery. All of the rats were trained to walk into a 50 × 7 cm^2^ wooden box, which contained track paper on the bottom. The left foot and toes of the rats were painted with nontoxic finger paint. A series of footprints was obtained. The variety of footprints reflected the degree of nerve injury and denervation. At least 5–10 measurable footprints were collected. The sciatic functional index (SFI) was calculated as equation (1):


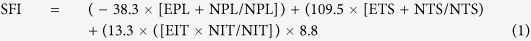


Print length (PL) is the distance from the heel to the top of the third toe, the intermediary toe spread (IT) is the distance between the second and the fourth toe, and toe spread (TS) is the distance from the first to the fifth toe. NPL, NTS, and NIT represent the PL, IT, and TS recorded from the non-operated foot, respectively. EPL, ETS, and EIT represent the PL, IT, and TS recorded from the operated foot, respectively^31^.

### Histochemical Staining of the Sciatic Nerve

After 2, 4, and 8 weeks, left sciatic nerves were harvested from rats in each group for histochemical staining. The nerves were fixed in 4% paraformaldehyde for 24 h, cryoprotected in 30% sucrose overnight at 4 °C, and then sectioned on a cryostat. The sections were attached to poly-lysine-precoated glass slides (Superfrost*/Plus microscope slides, Fisher Scientific) and prepared for staining. The tissues were washed with PBS three times for 15 min each, incubated with 0.1% Triton X-100 for 1 hour at room temperature, and blocking solution was applied (0.1% BSA), followed by incubation with the primary antibodies anti-NF200 protein mouse monoclonal antibody (Santa Cruz, 1:200) and anti-S100 protein rabbit monoclonal antibody (Santa Cruz, 1:200) at 4 °C overnight. The secondary antibodiesAlexa Fluor 488-labeled goat anti-mouse IgG (H + L) (1:500, Invitrogen) and Alexa Fluor 594-labeled goat anti-rabbit IgG (H + L) (1:500, Invitrogen) were applied to tissue samples for 2 hours at room temperature in the dark. This step was followed by washing in PBS. All of the slides were also stained with DAPI (1:500) at room temperature for 15 min away from light and were examined with a fluorescent microscope (BX-60; Olympus).

### Histological Analysis of Target Muscle

The gastrocnemius muscles from the operated limbs were harvested at the 8-week time point. Muscle specimens were fixed with formalin, embedded in paraffin, cut into 6 mm-thick sections and stained with hematoxylin and eosin. Random pictures from 3 fields of view were obtained from each sample and were analyzed with a Leica software package to measure the transverse section area of the muscle fibers. The percentage of muscle fiber area was calculated as muscle fiber area/total area.

### Statistical Analysis

All data are presented as the mean ± standard error of mean (SEM). One-way analysis of variance (ANOVA) was used to compare mean values with the SPSS11.0 software package (SPSS Inc, Chicago, IL). Values of *P* < 0.05 were considered statistically significant.

## Additional Information

**How to cite this article**: Ge, J. *et al*. Experimental immunological demyelination enhances regeneration in autograft-repaired long peripheral nerve gaps. *Sci. Rep.* **6**, 39828; doi: 10.1038/srep39828 (2016).

**Publisher's note:** Springer Nature remains neutral with regard to jurisdictional claims in published maps and institutional affiliations.

## Supplementary Material

Supplementary Information
