# Supplementary material for: Experimental immunological demyelination enhances regeneration in autograft-repaired long peripheral nerve gaps
Source: Sci Rep. 2016 Dec 23;6:39828. doi: 10.1038/srep39828 (PMC5180223; doi:10.1038/srep39828)
Supplement: Supplementary Information [file srep39828-s1.pdf]

Experimental immunological demyelination enhances regeneration in autograft-repaired long peripheral nerve gaps

Jun Ge*[1], Shu Zhu*[1], Yafeng Yang*[1], Zhongyang Liu*[1], Xueyu Hu[1], Liangliang Huang[1], Xin Quan[1], Meng Wang[2], Jinghui Huang[#1], YunQing Li[#3], and Zhuojing Luo[#1]

1, Institute of Orthopedics, Xijing Hospital, the Fourth Military Medical University, Xi'an 710032, PR China

2, General Political Department Hospital of PLA, Beijing 100120, PR China

3, The department of anatomy, the Fourth Military Medical University, Xi'an 710032, PR China

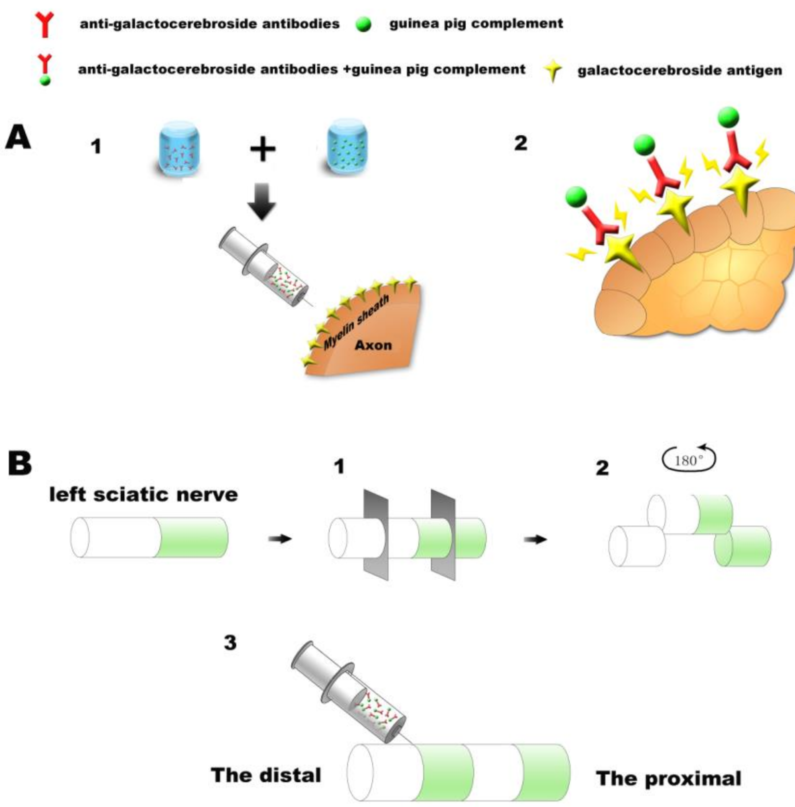

Fig 1s. Diagram of demyelinating process and surgical procedure. (A-1) The anti-galactocerebroside (Gal-C) antibody and guinea pig complement were compounded to the demyelinating agent with a ratio of 1:1. (A-2)The demyelinating agent effected on the myelin sheath in the injection area and induced an acute immunoreaction. (B-1) 12mm femoral nerve segment was excised. (B-2)The nerve segment was removed and reversed 180°.(B-3) after suturing the target segment back to the original position, 5μl agents (including demyelinating agent and placebo) were injected into epineurium in distal of the sciatic nerve graft. The sham group omitted this step.

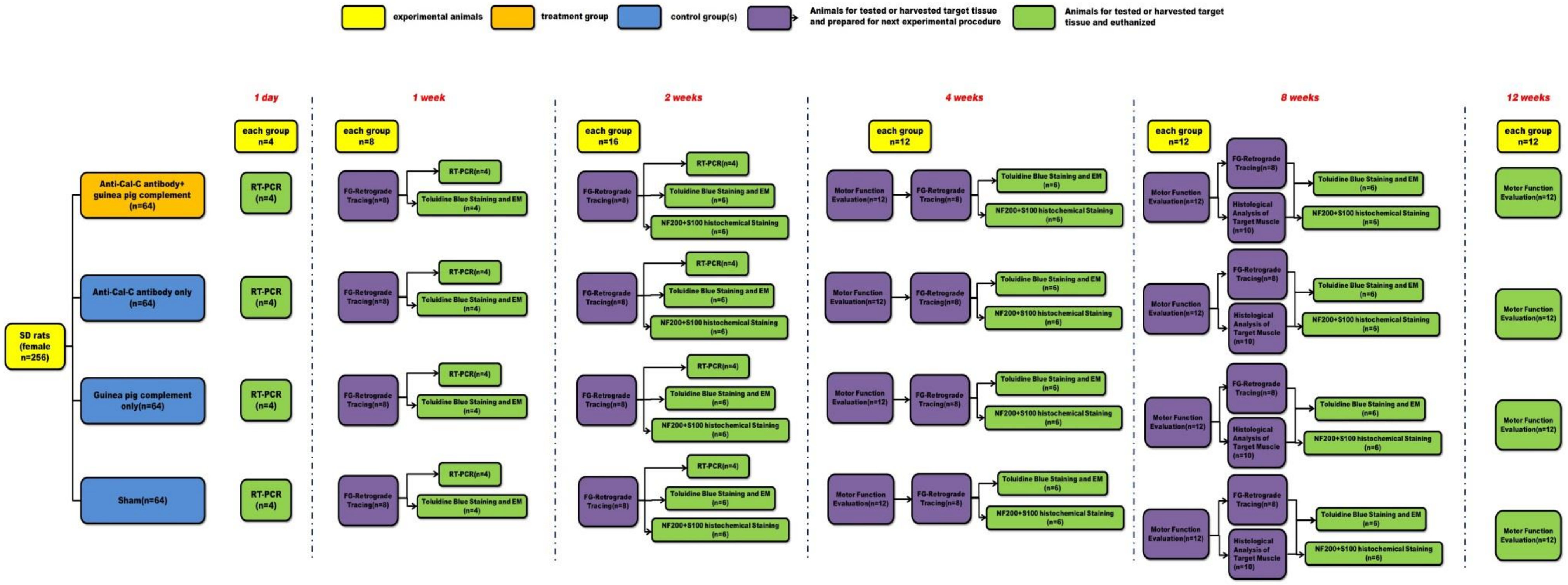

experimental animals
treatment group
control group(s)
Animals for tested or harvested target tissue and prepared for next experimental procedure
Animals for tested or harvested target tissue and euthanized
SD rats (female n=256)
Anti-Cal-C antibody+ guinea pig complement (n=64)
Anti-Cal-C antibody only (n=64)
Guinea pig complement only(n=64)
Sham(n=64)
1 day
each group n=4
RT-PCR (n=4)
1 week
each group n=8
FG-Retrograde Tracing(n=8)
RT-PCR(n=4)
Toluidine Blue Staining and EM (n=4)
2 weeks
each group n=16
FG-Retrograde Tracing(n=8)
RT-PCR(n=4)
Toluidine Blue Staining and EM (n=6)
NF200+S100 histochemical Staining (n=6)
4 weeks
each group n=12
Motor Function Evaluation(n=12)
FG-Retrograde Tracing(n=8)
Toluidine Blue Staining and EM (n=6)
NF200+S100 histochemical Staining (n=6)
8 weeks
each group n=12
Motor Function Evaluation(n=12)
FG-Retrograde Tracing(n=8)
Histological Analysis of Target Muscle (n=10)
Toluidine Blue Staining and EM (n=6)
NF200+S100 histochemical Staining (n=6)
12 weeks
each group n=12
Motor Function Evaluation(n=12)

Fig 2s. The experimental grouping and main process. 256 SD female rats were randomized into 4 groups: The anti-Cal-C antibody + guinea pig complement group, the anti-Cal-C antibody injection only group, the guinea pig complement injection only group and the sham group. All of the animals were received RT-PCR(in 1 day, 1 week, 2 weeks), FG-retrograde tracing(in 1 week, 2 weeks, 4 weeks and 8 weeks), toluidine blue staining(in 1 week, 2 weeks, 4 weeks and 8 weeks), electron microscopy (EM; in 1 week, 2 weeks, 4 weeks and 8 weeks), NF200+S100 histochemical staining(in 2 weeks, 4 weeks and 8 weeks), motor function evaluation (SFI; 4 weeks, 8 weeks and 12 weeks) and histological analysis of gastrocnemius tests(in 8 weeks) separately.

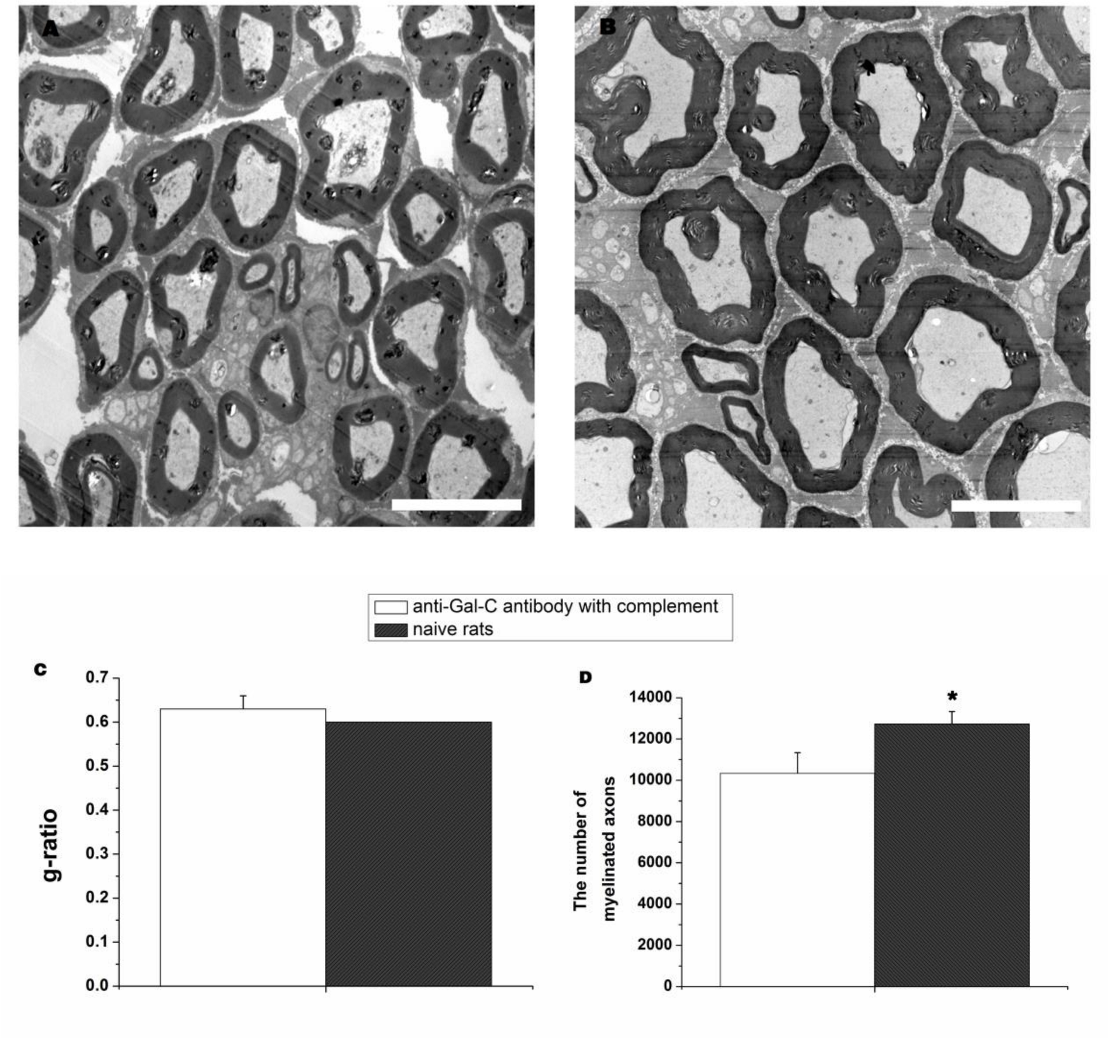

Fig 3s. Sciatic nerve electron microscopy photo of anti-Gal-C antibody with complement injection group in 8 weeks and the photo of naïve rats. (A) Nerve EM photo of anti-Gal-C antibody with complement injection showed a satisfactory recovery effect. (B) Naïve rats' nerve EM photo presented normal structures. (C) The g-ratio in anti-Gal-C antibody with complement injection group was approximate to naïve rats. (D) The quantification of the myelinated axons in demyelinating agent injection group was lower than naïve rats. * $P<0.05$ for the anti-Gal-C antibody and guinea pig complement injection group compare with naïve rats. Bar =10 μm.

Table 1s RT-PCR operating instructions

Factor	Forward Primer (5`-3`)	Reverse Primer (5`-3`)	Tm
IL-6	GCTGGGAGTCTTGTTC TGCTT	TGCTTCTATTTCCAC AACGCTTAC	F=60.95, R= 61.77
IL-10	GCTATGTTGCCTGCTC TTACTGG	TCTGGCTGACTGGG AAGTGG	F= 62.44, R= 63.74
IL-1β	CATAAGCCAACAAGTG GTATTCTCC	GGGTGTGCCGTCTT TCATC	F= 62.15, R=61.50
IFN-γ	TCCCGCCACCTATCTT TCAC	GCACCTCTCTTGCTT CCAGTTT	F= 62.30, R= 62.15
BDNF	GCCCAACGAAGAAAAC CATAA	CCAGCAGAAAGAGC AGAGGAG	F= 61.18, R=61.73
NGF	TTTTGCCTTTGCCTGG TTTC	GTTGATTGGCTGTG TCCCTTTAC	F= 62.35, R=61.97
